# Effects of stress-related neuromodulators on amygdala and hippocampus resting state functional connectivity

**DOI:** 10.1177/02698811241260972

**Published:** 2024-06-20

**Authors:** Catarina Rosada, Renée Lipka, Sophie Metz, Christian Otte, Hauke Heekeren, Katja Wingenfeld

**Affiliations:** 1 Department of Psychiatry, Charité Universitätsmedizin Berlin, Campus Benjamin Franklin, Berlin, Germany; 2 Berlin School of Mind and Brain, Humboldt Universität zu Berlin, Berlin, Germany; 3 Berlin Institute of Health, Institute of Medical Psychology, Charité Universitätsmedizin Berlin, Humboldt Universität zu Berlin, Berlin, Germany; 4 DZPG (German Center for Mental Health), Berlin, Germany; 5 Universität Hamburg, Hamburg, Germany

**Keywords:** Stress, hydrocortisone, yohimbine, amygdala, hippocampus, resting state functional connectivity

## Abstract

**Background:**

The human stress response is characterized by increases in neuromodulators, including norepinephrine (NE) and cortisol. Both neuromodulators can enter the brain and affect neurofunctional responses. Two brain areas associated with stress are the amygdala and the hippocampus. The precise influence of NE and cortisol on the amygdala and hippocampal resting state functional connectivity (RSFC) is poorly understood.

**Aims:**

To investigate the influence of NE and cortisol on the amygdala and hippocampal RSFC.

**Methods:**

We recruited 165 participants who received 10 mg yohimbine and/or 10 mg hydrocortisone in a randomized, placebo-controlled design. With seed-based analyses, we compared RSFC of the hippocampus and amygdala separately between the three groups that received medication versus placebo.

**Results:**

We found no differences between yohimbine and placebo condition or between hydrocortisone and placebo condition regarding amygdala or hippocampal FC. Compared with placebo, the yohimbine/hydrocortisone condition showed increased amygdala and hippocampal RSFC with the cerebellum. Also, they had increased hippocampal RSFC with the amygdala and cerebral white matter.

**Discussion:**

The group with elevated NE and cortisol showed significantly increased RSFC between the amygdala, hippocampus, and cerebellum compared to placebo. These three brain areas are involved in associative learning and emotional memory, suggesting a critical role for this network in the human stress response. Our results show that NE and cortisol together may influence the strength of this association. Compared to placebo, we found no differences in the groups receiving only one medication, suggesting that increasing one neuromodulator alone may not induce differences in neurofunctional responses. The study procedure has been registered at clinicaltrials.gov (ID: NCT04359147).

## Introduction

In response to stress, a fine-tuned neuroendocrinological interplay is initiated that involves the release of numerous neuromodulators that regulate neurological and physiological processes, which, in turn, regulate behavior (Romero and Butler, 2007; Russell and Lightman, 2019). In this study, we focused on two neuromodulators: the monoamine norepinephrine (NE) and the steroid cortisol. At the neurological level, we focused on the amygdala and the hippocampus. NE is released by the adrenal glands immediately after stress exposure as part of sympathetic nervous system (SNS) activation (Joëls, 2018; Joëls and Baram, 2009). Cortisol follows a natural circadian rhythm, with levels peaking in the early morning and decreasing throughout the day (Joëls, 2018). In response to stress, cortisol is additionally released through the hypothalamic–pituitary–adrenal (HPA) axis. In the brain, cortisol binds to two different receptors: glucocorticoid receptors (GRs) and mineralocorticoid receptors (MRs) (Groeneweg et al., 2012; Herman et al., 2016). MRs are mainly located in the limbic system and have a high affinity for glucocorticoids (cortisol); therefore, MRs are already occupied in non-stressful situations (Groeneweg et al., 2012). GRs are distributed throughout the brain and have a lower affinity for glucocorticoids, so they are only occupied when cortisol levels are high. NE acts rapidly (within minutes) to influence physiological and behavioral responses, for example, it promotes alertness, vigilance, and mobilization of energy resources (Joëls and Baram, 2009). Cortisol has similarly rapid non-genomic effects and is primarily expressed via MR activation in the hippocampus. These early synergistic effects of NE and cortisol are critical for immediate fight-or-flight responses and the rapid consolidation of emotional memories during stress. The hippocampus and amygdala, rich in NE and MR receptors, are critical for these processes. In addition, cortisol exerts slower genomic effects, primarily through the activation of glucocorticoid receptors (GRs) (Joëls, 2018). GRs initiate a feedback signal that terminates the stress response. After termination, GRs are involved in restoring homeostasis and contextualizing emotional memories. For the rapid consolidation of emotional memories and the contextualization of emotional memories, noradrenergic activations of the amygdala collaborate with cortisol-mediated hippocampal processes (Atucha et al., 2017; Van Ast et al., 2014).

While a functioning stress response allows us to respond appropriately to stressors and return to homeostasis, dysregulations of this system have been associated with somatic and psychiatric disorders (Chrousos, 2009). Stress-related disorders have been associated with endocrinological dysregulations, such as hypercortisolism in major depressive disorder (MDD) and hypocortisolism in posttraumatic stress disorder (PTSD) (Wingenfeld & Wolf, 2014), and structural and functional alterations of the hippocampus and amygdala (Drevets, 2003; Henigsberg et al., 2019). One prominent approach to study the brain in relation to stress and stress-related disorders is examining the brain’s blood oxygen level-dependent (BOLD) signal during rest (Fox, 2010). Identifying patterns of correlation in these spontaneous fluctuations has been termed resting state functional connectivity (RSFC).

Several studies have examined the stress-related changes in RSFC of the amygdala and hippocampus. Immediately after stress exposure, amygdala RSFC increased with brain regions of the salience network (SN; dorsal anterior cingulate cortex (ACC) and anterior insula) compared to a control group (Van Marle et al., 2010). The SN is involved in reorienting attention, mobilizing resources, and responding to threats (Hermans et al., 2014) which supports the notion of increased attention and mobilization immediately after stress exposure. One hour after stress induction, amygdala RSFC increased with regions of the default mode network (DMN; posterior cingulate cortex (PCC), precuneus, and medial prefrontal cortex (PFC)) compared to a control group (Veer et al., 2011). The DMN is involved in self-referential cognitive processes and memory (Raichle, 2015). The finding suggests the involvement of the amygdala in autobiographical memory processes during stress recovery. Interestingly, the strength of the relationship between the amygdala and medial PFC RSFC was mediated by cortisol—the higher the baseline cortisol, the stronger the subsequent negative functional connectivity (FC) between the two regions (Veer et al., 2012). Following studies further examined the differences between cortisol responders and non-responders in RSFC after stress exposure. Cortisol responders showed increased amygdala RSFC with the right parahippocampal gyrus and medial PFC, and reduced amygdala RSFC with the ventrolateral PFC, ventral PCC, culmen, and cuneus immediately after stress exposure compared to RSFC before stress exposure (Quaedflieg et al., 2015). Thirty minutes after stress exposure, cortisol responders showed increased RSFC between the amygdala and hippocampal areas and decreased RSFC between the amygdala and the dorsolateral PFC, dorsal ACC, and culmen (Quaedflieg et al., 2015). For the hippocampus, increased RSFC was reported immediately after stress exposure with the amygdala and medial temporal lobe areas, a pattern that returned to baseline 90 min after stress exposure (Vaisvaser et al., 2013). Taken together, the findings suggest a dynamic pattern of RSFC in individuals with cortisol response to psychosocial stress: Immediately after stress exposure, RSFC appears to increase between the amygdala and areas associated with threat detection (dorsal ACC, anterior insula, parahippocampal gyrus), and between the amygdala and the hippocampus. Recovery from stress appears to be associated with increased RSFC between the amygdala, hippocampus, and other brain areas of the DMN (PCC, medial PFC, precuneus), possibly supporting memory processes that are important for future adaptive behaviors.

The above-mentioned studies used psychosocial stressors, making it difficult to estimate the effects of NE and cortisol on amygdala/hippocampal RSFC. Studies using psychosocial stressors are important because they inform us about the natural stress response, but they only allow for correlational conclusions. With the current study, we want to increase knowledge about the causal effects of stress neuromodulators on individual brain areas by administering either exogenous NE or cortisol alone or both in combination before conducting a resting state scan. The knowledge could be used to better understand neuroendocrinological dysregulations and their effect on brain networks.

We found few studies examining the effects of exogenous cortisol on amygdala/hippocampal functions. One study found reduced activity in the hippocampus and amygdala, with a peak response minimum of 25–30 min after injection of 10 mg hydrocortisone compared to placebo (Lovallo et al., 2010). Another group examined the effect of 10 mg hydrocortisone on amygdala activity and FC during an emotional face-processing task (Henckens et al., 2010, 2012). They found a rapid decrease in amygdala activity and responsiveness to emotional stimuli 75 min after hydrocortisone intake compared to placebo (Henckens et al., 2010). They also reported decreased amygdala RSFC after hydrocortisone compared to placebo approximately 105 min after drug administration (Henckens et al., 2012). Positive amygdala RSFC was reduced in areas involved in initiating and maintaining the stress response (locus coeruleus, hypothalamus, hippocampus), and negative amygdala RSFC was reduced in areas involved in executive control (middle frontal gyrus, temporal gyrus). The findings suggest that cortisol inhibits amygdala and hippocampal activity and decouples the amygdala from the rest of the brain, perhaps supporting the normalization of brain function after stress exposure by ending the state of heightened vigilance associated with increased amygdala FC. So far, no study investigated the effects of exogenous noradrenaline on hippocampal or amygdala RSFC.

Taken together, the acute phase after stress exposure, when NE levels are particularly high, seems to be characterized by increased amygdala RSFC with areas that promote threat detection and sharpen an appropriate response to threat (Quaedflieg et al., 2015; Van Marle et al., 2010). In the intermediate phase, when NE and cortisol levels are high, there appears to be a shift with increased RSFC of the amygdala and hippocampus to support memory processes and promote future adaptive behaviors (Quaedflieg et al., 2015; Vaisvaser et al., 2013). The cortisol-specific findings suggest that elevated cortisol is associated with reduced FC of the amygdala and hippocampus (Henckens et al., 2010, 2012). This study aimed to investigate the effect of exogenous NE and/or cortisol on the amygdala and hippocampal RSFC. Participants received 10 mg yohimbine, a selective α2-adrenoceptor (AR) antagonist that regulates autonomic function (Tam et al., 2001) (to increase NE levels), and/or 10 mg hydrocortisone (to increase cortisol levels) prior to a resting state scan. In line with the literature, we expected the following for amygdala RSFC: increased positive RSFC with areas of the salience network (including ACC, AI) after yohimbine intake, increased positive RSFC with areas of the DMN (including the hippocampus, medial PFC) after yohimbine and hydrocortisone intake, and decreased RSFC after hydrocortisone intake compared to placebo. For the hippocampus, we expected increased RSFC with the amygdala after yohimbine intake, and after yohimbine and hydrocortisone intake, and decreased RSFC after hydrocortisone intake compared to placebo.

## Materials and methods

### Study design and procedure

We designed the study as a double-blind, randomized, placebo-controlled trial with four conditions: (A) yohimbine + placebo, (B) hydrocortisone + placebo, (C) hydrocortisone + yohimbine, and (4) placebo + placebo. For each participant, the study included two timepoints: (1) an interview to evaluate the exclusion criteria and a physical examination and (2) the functional magnetic resonance imagining (fMRI) session. Prior to the interview and physical examination, participants were informed about the study procedure and provided written informed consent. All fMRI sessions took place in the afternoon to control for diurnal cortisol variation. Participants received the first drug (yohimbine or placebo) 120 min before the resting state scan and the second drug (hydrocortisone or placebo) 105 min before the resting state scan. The scanner protocol consisted of several scans, including a decision-making task, an attention task, a resting state scan, and a structural scan. In this paper, we only used the resting state scan and the structural scan. For the resting state scan, we instructed participants to look at a fixation cross for 10 min. We then performed a structural T1-weighted scan for anatomical images. At five different timepoints, we collected saliva samples. The local ethics committee approved the study, and it was in accordance with the latest version of the Declaration of Helsinki.

### Participants

The study sample consisted of 167 healthy male subjects with a mean age of 25 years (SD = 4.25; ranging from 18 to 35). Exclusion criteria were left-handedness, body mass index over 30, self-reported history of physical or psychiatric illness, self-reported history of trauma, medication use, drug, or alcohol abuse, excessive sport (e.g., marathons), shift work, and fMRI contraindications (such as claustrophobia, non-removable magnetic material, tinnitus). Participants were recruited through universities, social media, and advertisements and received study credits or financial compensation of 60–90 Euros (depending on their performance in the decision-making task). Data from two participants had to be excluded from the current analyses due to artificial resting state data and premature termination of the experiment, resulting in 165 participants for data analyses.

### Saliva samples

We collected saliva samples with Salivette cotton swabs (Sarstedt) for the assessment of salivary cortisol and alpha amylase. After collection at room temperature, saliva samples were stored at −80°C until biochemical analysis. Saliva samples were analyzed at the Neurobiological Laboratory of the Charité Universitätsmedizin, Campus Benjamin Franklin. Free cortisol was analyzed using an adapted homogeneous time-resolved fluorescence resonance energy transfer-based competitive immunoassay. Inter-assay coefficients of variation were less than 10% and intra-assay coefficients of variation were less than 8%. The limit of detection for free cortisol was 0.2 nM (for a detailed description, see Duesenberg et al., 2016). Alpha amylase was evaluated by direct alpha-amylase assay using 2-chloro-4-nitrophenyl-a-D-maltotrioside.

### Statistical analyses

We performed all statistical analyses with IBM SPSS Statistics version 26 (IBM Armonk, NY, USA). Analyses of variance (ANOVAs) were performed to characterize the sample with age, and BMI as dependent variables and group as the independent variable. For categorical variables, we performed Chi-squared tests to compare groups in level of education, marital status, and smoking habits. For manipulation control, we performed a mixed ANOVA with treatment variable (yohimbine vs. hydrocortisone vs. yohimbine + hydrocortisone vs. placebo) as a between-subject factor, time as within-subject factor, and salivary cortisol and salivary alpha amylase as dependent variables. With Bonferroni-corrected planned comparisons, we examined which timepoints differed from the baseline for each group and which experimental groups differed from placebo at each timepoint.

### FMRI data acquisition

We used a 3 Tesla Siemens Magnetom TimTrio with a 32-channel head coil for the fMRI data acquisition. The resting state scans were collected with a T2-weighted echo planar imaging sequence with the subsequent parameters: repetition time (TR) = 2000 ms, flip angle = 70°, slice thickness = 3 mm, inter-slice gap = 3.3 mm, echo time (TE) = 30 ms, field of view = 192 mm, and voxel size = 3 × 3 × 3 mm^3^ with a total of 300 whole brain volumes. After the resting state scan, we acquired high-resolution structural T1-weighted images with the following parameters: TR = 1900 ms, flip angle = 9°, slice thickness = 1 mm, inter-slice gap = 0 mm, TE = 2.52 ms, a field of view = 256 mm, and voxel size = 1 × 1 × 1 mm^3^.

### FMRI data preprocessing

Imaging data were preprocessed and analyzed using the Oxford Centre for Functional MRI of the Brain (FMRIB) Software Library (FSL; Jenkinson et al., 2012). We first preprocessed the T1-weighted structural images using the fsl_anat processing script (https://fsl.fmrib.ox.ac.uk/fsl/fslwiki/fsl_anat). The script includes reorientation, bias-field correction, and skull stripping. For a more refined brain extraction, we used the Advanced Normalization Tools (ANTs) abpBrainExtraction script (v 0.5.6; Avants et al., 2009) and where necessary, manually edited the skull strip in freeview (v.6.0.0; https://surfer.nmr.mgh.harvard.edu/). After brain extraction, the anatomical T1-weighted data were segmented using FAST (Automated Segmentation Tool; https://fsl.fmrib.ox.ac.uk/fsl/fslwiki/FAST; Zhang et al., 2001). For linear and nonlinear registration to standard space, we used FLIRT (FMRIB’s Linear Image Registration Tool; https://fsl.fmrib.ox.ac.uk/fsl/fslwiki/FLIRT; Jenkinson et al., 2002; Jenkinson & Smith, 2001) and FNIRT (FMRIB’s Nonlinear Image Registration Tool; https://fsl.fmrib.ox.ac.uk/fsl/fslwiki/FNIRT; Jenkinson et al., 2012). To preprocess the T2-weighted resting-state functional data, we used BET (Brain Extraction Tool; https://fsl.fmrib.ox.ac.uk/fsl/fslwiki/BET; Smith, 2002) for skull stripping and bias field correction. We motion-corrected the functional data using MCFLIRT (Motion Correction FMRIB Linear Registration Tool; https://fsl.fmrib.ox.ac.uk/fsl/fslwiki/MCFLIRT; Jenkinson et al., 2002) and ICA-AROMA (Independent Component Analysis Automatic Removal of Motion Artifacts; https://fsl.fmrib.ox.ac.uk/fsl/fslwiki/OtherSoftware; Pruim et al., 2015). We then co-registered structural and functional data using BBR (Boundary-Based Registration; https://fsl.fmrib.ox.ac.uk/fsl/fslwiki/FLIRT_BBR; Greve & Fischl, 2009). For the registrations, we used the following standard space template: MNI152 T1 2 mm. An exploratory analysis of motion showed that the four experimental groups did not differ in motion (*F* (3,161) = 0.914, *p* = 0.436, η^2^ = 0.017)) and the motion was very low across the whole sample (M = 0.3 mm, SD = 0.56).

### FMRI data analysis

To examine RSFC of the amygdala and hippocampus after intake of hydrocortisone and/or yohimbine compared with placebo, we first extracted binary masks of the hippocampus and the amygdala from the Harvard-Oxford subcortical structural atlas, provided in FSL. To create the masks, we included voxels that belonged to the hippocampus/amygdala with at least 50% probability. We then extracted a time series of bilateral hippocampus and amygdala separately for every participant. In addition, we extracted white matter (WM) and cerebrospinal fluid (CSF) time series for each participant. To do this, we used the masks for WM and CSF created with FAST. We then ran a single-subject general linear model (GLM) for each ROI with the time series of the ROI (hippocampus or amygdala) as the regressor of interest and the time series of the WM and CSF as covariates using fsl_glm. This resulted in two connectivity maps for each subject, one for the hippocampus and one for the amygdala. The connectivity maps represent the FC between the ROI and other brain areas after regressing the effects of WM and CSF. For group analyses, we used FSL’s nonparametric permutation test randomize with 10,000 permutations and threshold-free cluster enhancement (TFCE) to correct for multiple comparisons (https://fsl.fmrib.ox.ac.uk/fsl/fslwiki/Randomise/UserGuide; Winkler et al., 2014). We conducted pairwise comparisons between each treatment group and placebo in both directions. As additional exploratory analyses, we performed the same analyses separately for the left and right amygdala and hippocampus.

## Results

### Sample characteristics: Demographics, salivary alpha amylase, and salivary cortisol

The characterization of our sample showed no group differences between the four groups on any of the demographics of interest. The demographics are presented in Table 1.

**Table 1.** Sample characteristics.

	Placebo (*N* = 42)	Yohimbine (*N* = 40)	Hydrocortisone (*N* = 41)	Yohimbine + hydrocortisone (*N* = 42)	Statistics
Age (*M*, *SD*)	25.36 (4.42)	26.10 (4.05)	24.71 (4.47)	24.88 (4.14)	*F* (3, 161) = 0.85; *p* = 0.463
BMI (*M*, *SD*)	23.70 (2.79)	23.17 (2.70)1 missing	23.22 (2.54)	23.47 (2.24)	*F*(3, 160) = 0.36; *p* = 0.777
Level of education					
German A-level	39 (92.9%)	38 (95.0%)	40 (97.6%)	40 (95.2%)	*x*^2^(3) = 0.998; *p* = 0.802
Other	3 (7.1%)	2 (5.0%)	1 (2.4%)	2 (4.8%)	
Smoking habits					
Smoking	10 (23.8%)	5 (12.8%)	7 (17.5%)	7 (16.7%)	*x*^2^(3) = 1.74; *p* = 0.629
Non-smoking	32 (76.2%)	34 (87.2%)1 missing	33 (82.5%)1 missing	35 (83.3%)	
Family status					
Single	37 (90.2%)	31 (77.5%)	37 (92.5%)	36 (85.7%)	*x*^2^(3) = 4.52; *p* = 0.210
Relationship	4 (9.8%)1 missing	9 (22.5%)	3 (7.5%)	6 (14.3%)	

BMI: body mass index; M: mean; SD: standard deviation.

For salivary alpha amylase, the repeated measures ANOVA revealed a significant main effect of time (*F*(3.44, 552.96) = 17.32, *p* < 0.001, *η*^2^ = 0.10), a significant main effect of treatment (*F*(3, 161) = 3.13, *p* = 0.027, *η*^2^ = 0.06), and a significant interaction between treatment and time (*F*(10.30, 552.96) = 2.74, *p* = 0.002, *η*^2^ = 0.05). Bonferroni-corrected pairwise comparisons revealed that compared to baseline, salivary alpha amylase was still elevated in the yohimbine conditions at the time point closest to our resting state scan (+155 min).

For salivary cortisol, we found a significant main effect of hydrocortisone (*F*(1, 161) = 97.87, *p* < 0.001, *η*^2^ = 0.37) and a significant interaction between hydrocortisone and time (*F*(1.64, 265.07) = 74.77, *p* < 0.001, *η*^2^ = 0.31). Bonferroni-corrected pairwise comparisons showed that compared to baseline, salivary cortisol was still elevated in the hydrocortisone conditions at the time point closest to our resting state scan (+155 min). We found no significant main effect of yohimbine on salivary cortisol (*F*(1, 161) = 0.04, *p* = 0.83, *η*^2^ = 0.00). Group means and standard deviations for salivary alpha amylase and cortisol are presented in Table 2.

**Table 2.** Salivary alpha amylase and cortisol.

	Placebo (*N* = 42)	Yohimbine (*N* = 40)	Hydrocortisone (*N* = 41)	Yohimbine + hydrocortisone (*N* = 42)
Alpha amylase (*M*, *SD*)				
Baseline	130.56 (84.91)	153.59 (83.28)	120.46 (90.42)	143.83 (110.92)
Baseline + 15 min	127.88 (81.95)	151.89 (80.44)	123.82 (90.85)	134.15 (101.32)
Baseline + 75 min	153.48 (106.71)	207.08 (102.75)	134.79 (91.91)	185.50 (110.16)
Baseline + 155 min	144.16 (98.96)	186.41 (100.98)	117.80 (87.01)	177.07 (114.77)
Baseline + 170 min	145.50 (112.06)	189.83 (106.70)	110.39 (96.66)	178.76 (114.64)
Cortisol (*M*, *SD*)				
Baseline	4.15 (3.19)	5.08 (3.09)	4.50 (3.14)	4.24 (4.08)
Baseline + 15 min	3.69 (3.35)	4.47 (3.12)	4.72 (3.52)	3.62 (2.98)
Baseline + 75 min	5.52 (7.23)	6.07 (4.79)	27.81 (16.86)	28.45 (22.82)
Baseline + 155 min	3.08 (3.77)	3.37 (2.21)	16.66 (10.95)	17.44 (15.30)
Baseline + 170 min	3.05 (3.52)	3.52 (3.15)	16.03 (11.86)	14.97 (9.56)

M: mean; SD: standard deviation.

### ROI analyses: Amygdala and hippocampal RSFC

**Figure 1.**
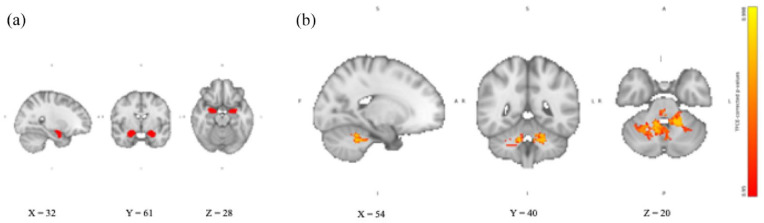
Amygdala RSFC after combined intake of yohimbine and hydrocortisone compared with placebo. (a) Amygdala seed region extracted from Harvard-Oxford subcortical structural atlas in FSL overlaid on the 2 mm isotropic 152-MNI standard space brain. (b) Amygdala RSFC TFCE-corrected *p*-value maps showing increased amygdala RSFC with cerebellar areas after intake of yohimbine, and hydrocortisone compared with placebo. Results are overlaid on the 2 mm isotropic 152-MNI standard space brain. R: right; L: left; A: anterior; P: posterior; S: superior; I: inferior; TFCE: threshold-free cluster enhancement.

For the amygdala, we found no significant differences in the RSFC between the two groups receiving either hydrocortisone or yohimbine alone and placebo (in either direction). Similarly, exploratory analysis of the left and right amygdala showed no differences in RSFC between the two groups receiving hydrocortisone or yohimbine alone and placebo. Compared to placebo, the group receiving yohimbine and hydrocortisone together showed significantly increased amygdala RSFC in a cluster located in the cerebellum (see Figure 1). The cluster was identified as the cerebellum using the MNI structural atlas in FSL. The exploratory analyses showed that both the left and right amygdala significantly increased RSFC with the cerebellum.

**Figure 2.**
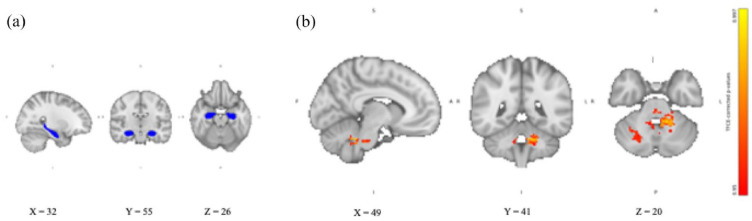
Hippocampal RSFC after combined intake of yohimbine and hydrocortisone compared with placebo. (a) Hippocampal seed region extracted from Harvard-Oxford subcortical structural atlas in FSL overlaid on the 2 mm isotropic 152-MNI standard space brain. (b) Hippocampal RSFC TFCE-corrected *p*-value maps showing increased hippocampal RSFC with cerebellar areas after intake of yohimbine and hydrocortisone compared with placebo. Results are overlaid on the 2 mm isotropic 152-MNI standard space brain. R: right; L: left; A: anterior; P: posterior; S: superior; I: inferior; TFCE: threshold-free cluster enhancement.

**Figure 3.**
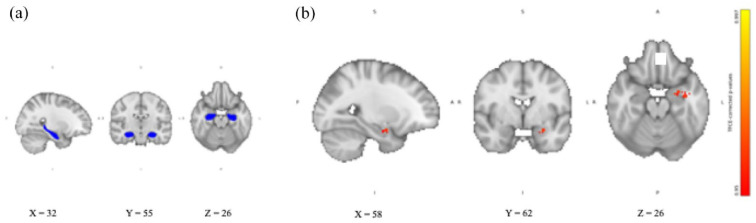
Hippocampal RSFC after combined intake of yohimbine and hydrocortisone compared with placebo. (a) Hippocampal seed region extracted from Harvard-Oxford subcortical structural atlas in FSL overlaid on the 2 mm isotropic 152-MNI standard space brain. (b) Hippocampal RSFC TFCE-corrected *p*-value maps showing increased hippocampal RSFC left amygdala after intake of yohimbine, and hydrocortisone compared with placebo. Results are overlaid on the 2 mm isotropic 152-MNI standard space brain. R: right; L: left; A: anterior; P: posterior; S: superior; I: inferior; TFCE: threshold-free cluster enhancement.

For the hippocampus, we found no significant differences in the RSFC between the two groups receiving hydrocortisone alone or yohimbine alone and placebo (in either direction). Similarly, exploratory analysis of the left and right hippocampus separately showed no differences in RSFC between the two groups receiving hydrocortisone alone or yohimbine alone and placebo. Compared to placebo, the group receiving the combination of yohimbine and hydrocortisone showed significantly increased RSFC with the cerebellum (see Figure 2), the left amygdala (see Figure 3), and the left cerebral WM. The cerebellum was identified using the MNI structural atlas in FSL, and the amygdala and cerebral WM were identified using the Harvard-Oxford subcortical structural atlas in FSL. The exploratory analyses showed that only the left hippocampus significantly increased RSFC with the cerebellum.

An overview of the clusters with significant RSFC contrasts is shown in Table 3.

**Table 3.** Clusters with significant RSFC contrast: Yohimbine + hydrocortisone > placebo.

Cluster location	Size (voxels)	Peak *p*-values	MNI peak voxel coordinates		
			*x*	*y*	*z*
Amygdala					
Cerebellum	751	0.011	54	40	20
Left amygdala					
Cerebellum	464	0.011	54	40	20
Right amygdala					
Cerebellum	744	0.009	39	39	19
Hippocampus					
Cerebellum	313	0.013	49	41	20
Right VI cerebellum	62	0.036	34	30	21
Right IX cerebellum	33	0.040	42	38	20
Left amygdala	30	0.036	58	62	26
Left cerebral WM	14	0.036	63	58	26
Left hippocampus					
Cerebellum	116	0.022	55	41	19

TFCE-cluster corrected *p*-values (threshold: *p* < 0.05). Clusters were identified using the MNI structural atlas (cerebellum), the probabilistic cerebellar atlas (right VI cerebellum, right IX cerebellum), and the Harvard-Oxford subcortical structural atlas (left amygdala, left cerebral WM). Only clusters larger than ten voxels are displayed.

WM: white matter.

## Discussion

This study aimed to investigate the effects of stress-related neuromodulators NE and cortisol on the amygdala and hippocampal RSFC. We administered 10 mg yohimbine (to increase NE levels) and/or 10 mg hydrocortisone (to increase cortisol) and performed a resting state scan to measure amygdala and hippocampal RSFC. During the resting state scan, salivary alpha amylase, an indicator of NE activation, was still elevated in both yohimbine-treated groups compared to the placebo and hydrocortisone groups. Salivary cortisol was still elevated in both groups receiving hydrocortisone compared to the placebo and yohimbine groups. Thus, the pharmacological manipulation was effective. The RSFC data analyses show the following: First, we found no significant differences in the RSFC of the amygdala or hippocampus with any other brain area and in either direction (increased or decreased RSFC) in the group receiving yohimbine alone compared to placebo. Second, we found no significant differences in the RSFC of the amygdala or hippocampus with any other brain area and in either direction (increased or decreased RSFC) in the group receiving hydrocortisone alone compared to placebo. Third, in the group receiving both substances, we found significantly increased amygdala and hippocampal RSFC with cerebellar regions and increased hippocampal RSFC with the left amygdala and the left cerebral WM compared to placebo. The three group comparisons are discussed in detail below.

### RSFC after noradrenergic stimulation

We found no differences in the amygdala or hippocampal RSFC between the yohimbine and the placebo group. The finding is inconsistent with our hypotheses. We expected increased positive amygdala RSFC with areas of the salience network (e.g., anterior insula and ACC) and increased positive hippocampal-amygdala RSFC after yohimbine intake compared to placebo. The hypothesis was based on previous studies that reported increased RSFC within these brain circuits immediately after stress exposure, a period when the sympathetic nervous system is activated and NE levels are particularly high (Quaedflieg et al., 2015; Vaisvaser et al., 2013; Van Marle et al., 2010). Compared to the placebo, the yohimbine group had significantly increased salivary alpha amylase during the resting state scan. Salivary alpha amylase has been proposed as a noninvasive biomarker of noradrenergic activation (Nater & Rohleder, 2009). Thus, it appears that we could increase noradrenergic activation with the administration of yohimbine. However, we did not find the same neurofunctional response that has been reported after stress exposure. Immediately after stress exposure, the salience network, including the amygdala, is recruited for behavioral tasks such as thread detection, attentional reorientation, and resource mobilization (Hermans et al., 2014). At the same time, the hippocampus and amygdala are functionally linked to support associative learning and the formation of emotional memories (Janak & Tye, 2015). Both processes are important for acute and future adaptive behavior in response to stress. To our knowledge, this is the first study to investigate the effects of an α2-adrenoreceptor antagonist like yohimbine on RSFC. Although our findings suggest that elevated NE levels alone may not be sufficient to trigger the neurofunctional response associated with an acute stress response, others convincingly demonstrated that noradrenergic activation is essential for the recruitment of the salience network following acute stress exposure (Hermans et al., 2011). In their placebo-controlled study, they administered drugs to either reduce noradrenergic activation or cortisol elevation following stress exposure and showed that only reducing noradrenergic activation resulted in reduced FC of the salience network compared to placebo (Hermans et al., 2011). A possible explanation for our finding may be that the difference in noradrenergic activation between the yohimbine and placebo groups was not large enough at the time of the resting state scan. Future studies could further investigate the effect of yohimbine administration on amygdala/hippocampal RSFC by measuring RSFC at a time closer to drug administration when noradrenergic activation may be stronger.

### RSFC after cortisol elevation

We found no differences in the amygdala or hippocampal RSFC between the hydrocortisone and the placebo group. Again, the result is inconsistent with our hypotheses. Based on previous studies in which hydrocortisone was administered before a resting state scan, we expected functional decoupling of the amygdala and hippocampus from the rest of the brain (Henckens et al., 2012; Lovallo et al., 2010). One study has shown that hydrocortisone administration results in decreased activity of the amygdala and hippocampus, which peaks approximately half an hour after administration (Lovallo et al., 2010). For the amygdala, positive RSFC decreased with brain regions involved in the initiation and regulation of the stress response, such as the locus coeruleus, hypothalamus, and hippocampus, approximately 100 min after administration (Henckens et al., 2012). Reduced negative RSFC was also reported between the amygdala and brain regions involved in executive control (middle frontal and middle temporal gyrus) at this time point. The authors suggest that cortisol may play an important role in restoring homeostasis after stress by reducing the influence of emotional brain processes (involving the amygdala and hippocampus) and thereby supporting executive control processes (involving frontal brain areas) (Henckens et al., 2012). Importantly, after entering the brain, cortisol binds to two different receptors: MRs and GRs (Joëls, 2018). While MRs are primarily located in the limbic system and have a high affinity for glucocorticoids, GRs are more ubiquitously distributed throughout the brain and have a lower affinity for glucocorticoids. MRs are rapidly occupied even under non-stressful conditions and are involved in the acute stress response when emotional processes are promoted. GRs are fully activated only under stressful conditions and are involved in more genomic effects of cortisol and the termination of the stress response, promoting executive functions. In our study, the mean time between hydrocortisone administration and the resting state scan was 112 min (ranging from a minimum of 98 min to a maximum of 150 min). The time point that we investigated would match the phase, when executive functions are promoted, suggesting a functional decoupling of the limbic system. However, it remains difficult to disentangle the slow and fast effects of cortisol on brain processing. In addition, the effect of the same dose of hydrocortisone may differ, for example, depending on an individual’s basal cortisol concentration, which is influenced by both genetic and environmental factors (Bartels et al., 2003; Zänkert et al., 2019). Although we attempted to control for differences in cortisol wake response by performing all fMRI sessions in the afternoon, we cannot exclude the possibility that participants responded differently to hydrocortisone administration and showed different neurofunctional response patterns, which may have even counterbalanced each other. However, this is highly speculative and should be investigated in future studies by relating individual responses to hydrocortisone to amygdala and hippocampal FC patterns.

### RSFC after noradrenergic activation and cortisol elevation

Compared to placebo, the group receiving both yohimbine and hydrocortisone had increased amygdala and hippocampal RSFC with areas of the cerebellum and increased hippocampal RSFC with the left amygdala and the left cerebral WM. For the amygdala, this effect was found bilaterally, while for the hippocampus, the effect was driven by the left hippocampus. This is only partly consistent with our hypotheses. We expected that increasing NE and cortisol would result in increased RSFC between the hippocampus and amygdala and between the amygdala and other areas of the DMN (Quaedflieg et al., 2015; Vaisvaser et al., 2013). We did find increased hippocampal-amygdala RSFC, which supports the idea of an activated network that has been implicated in associative learning such as fear conditioning and emotional memory formation (Janak & Tye, 2015; Yang & Wang, 2017). We did not hypothesize increased FC between the hippocampus/amygdala and the cerebellum or cerebral WM. The increased RSFC between the hippocampus and cerebral WM appears to be an artifact, as we attempted to control for WM effects in our analyses. However, the robust associations of the amygdala and the hippocampus with the cerebellum are very interesting, as they are consistent with the idea that increased NE, along with increased cortisol, affects a network responsible for stress-related learning and memory processes. While traditionally, the cerebellum has mainly been associated with motor functions and coordination, recent studies have proposed a central role for the cerebellum in the neurofunctional stress response (Moreno-Rius, 2019). The cerebellum is associated with the noradrenergic system, and its large number of GRs and connections to the hypothalamus and hippocampus suggest a role in the regulation of the HPA axis (Schambra et al., 2005; Schutter, 2012). Neurofunctionally, the cerebellum is connected to both limbic and cortical structures, and behaviorally, many studies support the role of the cerebellum in cognitive and emotional processes (Schutter and Van Honk, 2005; Wagner and Luo, 2020). For example, the cerebellum seems to play a role in motor, cognitive, and emotional associative learning like fear conditioning (Lange et al., 2015; Timmann et al., 2010). In addition, the cerebellum seems to be activated in emotional memory encoding in conjunction with the hippocampus and amygdala (Fastenrath et al., 2022). The authors thus suggest the involvement of all three brain areas (hippocampus, amygdala, and cerebellum) in a network involved in emotional memory enhancement. Emotional memory, in turn, is affected by stress and stress hormones (De Quervain et al., 2017; Wolf, 2008). Combining these findings with the current results, we can suggest that the simultaneous elevation of NE and cortisol may promote a brain network, including the amygdala, hippocampus, and cerebellum, that may behaviorally promote emotional memory processes. The finding that only the left hippocampus appears to be involved in this network is consistent with previous studies that found a functional split within the hippocampus: while the right hippocampus appears to be involved in spatial memory, the left hippocampus appears to be more important for episodic and emotional memory (Burgess et al., 2002). Future research could combine our study design with an emotional memory task to determine whether increased RSFC between the hippocampus/amygdala and cerebellum after stress hormone elevation results in emotional memory changes.

### Strengths and limitations

To our knowledge, this is the first study to examine the effects of exogenous NE and cortisol administration on amygdala and hippocampal RSFC. Compared to studies using naturalistic stressors, our study design allowed us to examine more causal relationships between each stress neuromodulator and RSFC of individual brain regions, as there is a reduced risk that results are confounded by individual differences in psychological aspects of the stress response (e.g., interpretation of the situation), thereby increasing the internal validity. However, because pharmacological manipulation of stress-related neuromodulators is not equivalent to the natural human stress response, construct validity is compromised by the study design. For example, many more neuromodulators, including dopamine, serotonin, and corticotropin-releasing hormone, play an important role in the naturalistic stress response (Joëls & Baram, 2009). In the current study, we did not assess these neuromodulators, so they may have influenced the present results. Also, real-life stressors must be unpredictable and uncontrollable, which is not the case with the pharmacological manipulation we used in the present study (Sandi, 2013). However, as psychological and endocrinological measures of stress are highly correlated, both measures seem to be indicators of the same construct (Schlotz et al., 2008). The pharmacological elevation of NE and cortisol can therefore be interpreted in the context of stress research, but we must be cautious when relating our findings to stressful situations in real life.

Another strength of the study is the carefully selected sample. We excluded individuals with, for example, a history of childhood trauma, physical or mental disorders, medication use, shift work, and excessive sport as those factors may affect baseline levels of stress hormones (Zänkert et al., 2019). However, the carefully selected sample limits our ability to make inferences about the general population. Most importantly, results are not transferable to women, because many studies have shown sex differences in the neuroendocrinological stress response (Bangasser and Valentino, 2014). Future studies should replicate the study with a female sample. The exclusion of individuals with a history of traumatic experiences is also relevant, as early life stress has been reliably associated with the dysregulation of stress-regulating systems (Agorastos et al., 2018).

Importantly, the data were acquired in a larger fMRI study that included decision-making and an emotional attention task between the pharmacological manipulation and the resting state scan. Thus, we cannot exclude the possibility that the paradigms confounded the association between stress neuromodulators and amygdala/hippocampal RSFC (Cole, 2010). While we pre-registered the analyses for the decision-making and the attentional task, the resting state analyses were not preregistered. Thus, analyses may be regarded as rather exploratory and should be replicated in future studies.

Another limitation concerns cortisol measurements. As mentioned above, cortisol follows a circadian rhythm with high levels in the morning and decreasing levels throughout the day (Joëls, 2018). In addition, the release of cortisol is pulsatile, resulting in multiple peaks throughout the day. In our sample, we attempted to control for circadian variations in cortisol by performing all measurements in the afternoon. However, baseline measurements were taken within a time frame of approximately 3 h, and a group analysis showed that significantly more participants in the placebo group were tested later in the afternoon than in the other three groups. This could mean that the placebo group had lower levels of endogenous cortisol than the other groups, which could have potentially affected group differences. We cannot exclude the possibility that the study results were influenced by the timing of the fMRI measurement. In addition, studies have shown that measuring serum cortisol with salivary cortisol has its limitations: while low levels of serum cortisol are often undetectable in salivary cortisol, oral hydrocortisone can lead to falsely high salivary cortisol levels (Debono et al., 2016; Imamovic et al., 2023). Alternatively, salivary cortisone measurements have been suggested as a more reliable measure of serum cortisol (Debono et al., 2016). However, within the current study, the saliva samples serve only as a manipulation control, and we did not aim to make statements about serum cortisol levels. Future studies could replicate the present study, including stricter control of the timing of fMRI measurements and salivary cortisone measurements, to investigate the effect of actual cortisol levels on RSFC more precisely.

Finally, it is important to note that our results are limited to the amygdala- and hippocampus-based networks because we used a seed-based approach with the amygdala and hippocampus as ROIs. Although the amygdala and hippocampus play an important role in stress-related processes, other brain areas such as the prefrontal cortex are also involved (McEwen et al., 2016). In addition, we had to use different atlases in FSL to identify the clusters because the Harvard-Oxford subcortical structural atlas, which we used for the amygdala/hippocampus masks, did not identify the cerebellar brain areas. Future studies could try to replicate the current findings using an atlas that can identify the amygdala/hippocampus and the cerebellum, and further extend the knowledge by selecting different brain areas as ROIs (e.g., cerebellum, prefrontal cortex).

## Conclusion

In the current study, we showed that pharmacological manipulation of a single neuromodulator (NE or cortisol) did not result in changes in the amygdala or hippocampal RSFC compared to placebo. Based on earlier studies, we expected to find increased positive amygdala RSFC with areas of the salience network (e.g., anterior insula and ACC) and increased positive hippocampal-amygdala RSFC after yohimbine intake compared to placebo, and decreased RSFC of the amygdala and hippocampus after hydrocortisone intake compared to placebo. The present results suggest that increasing a single neuromodulator may not be sufficient to elicit neurofunctional responses comparable to those elicited by real-life stressors. However, other factors, for example, the timing of the resting state scan in relation to the pharmacological manipulation may also have influenced the results. Future studies should replicate the study after considering the limitations of the current study. We further showed that pharmacological manipulation of both neuromodulators (NE and cortisol) together resulted in increased amygdala and hippocampal RSFC with cerebellar areas and increased hippocampal RSFC with the amygdala. Again, the finding was not in line with what we expected based on studies that examined the naturalistic response to stress. The current finding suggests that an increase in both neuromodulators may activate a functional brain network including the amygdala, the hippocampus, and the cerebellum that may be associated with important behavioral stress responses such as fear conditioning and emotional memory. The current study thus supports the idea that noradrenergic activation and cortisol are important for the emotional memory processes that are part of the stress response. Also, the current study results support the paradigm shift to include the cerebellum in stress-related research. Future research should further investigate the relationship between stress, FC between the cerebellum and the limbic system, and memory processes, for example, by testing the a priori hypothesis that the synergy of noradrenergic activation and cortisol recruits this specific network, including the hippocampus, amygdala, and cerebellum, and by investigating whether and how the activation of this network translates into behavioral effects.

## Supplemental Material

sj-doc-2-jop-10.1177_02698811241260972 – Supplemental material for Effects of stress-related neuromodulators on amygdala and hippocampus resting state functional connectivity Supplemental material, sj-doc-2-jop-10.1177_02698811241260972 for Effects of stress-related neuromodulators on amygdala and hippocampus resting state functional connectivity by Catarina Rosada, Renée Lipka, Sophie Metz, Christian Otte, Hauke Heekeren and Katja Wingenfeld in Journal of Psychopharmacology

sj-docx-1-jop-10.1177_02698811241260972 – Supplemental material for Effects of stress-related neuromodulators on amygdala and hippocampus resting state functional connectivity Supplemental material, sj-docx-1-jop-10.1177_02698811241260972 for Effects of stress-related neuromodulators on amygdala and hippocampus resting state functional connectivity by Catarina Rosada, Renée Lipka, Sophie Metz, Christian Otte, Hauke Heekeren and Katja Wingenfeld in Journal of Psychopharmacology
