# Supplementary material for: Effects of stress-related neuromodulators on amygdala and hippocampus resting state functional connectivity
Source: J Psychopharmacol. 2024 Jun 20;38(7):604–14. doi: 10.1177/02698811241260972 (PMC11290027; doi:10.1177/02698811241260972)
Supplement: sj-doc-2-jop-10.1177_02698811241260972 – Supplemental material for Effects of stress-related neuromodulators on amygdala and hippocampus resting state functional connectivity [file sj-doc-2-jop-10.1177_02698811241260972.doc]

**
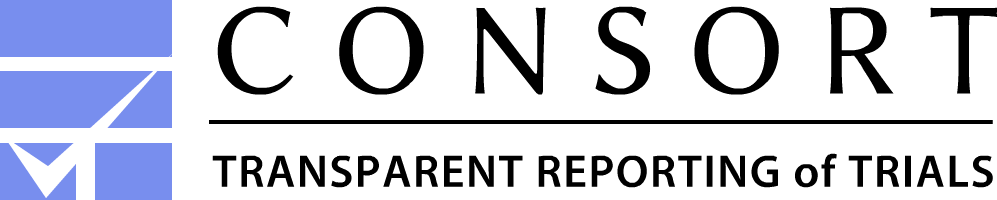
**

**CONSORT Flow Diagram**

**Allocation**

**Analysis**

**Follow-Up**

**Enrollment**

Assessed for eligibility (n= 167)

164

Excluded (n= / )

♦  Not meeting inclusion criteria (n= )

♦  Declined to participate (n= )

♦  Other reasons (n= )

Analysed (n= 165)
♦ Excluded from analysis (give reasons) (n=2, premature termination of experiment, artificial data)

Lost to follow-up (give reasons) (n= )

Discontinued intervention (give reasons) (n= )

Allocated to intervention (n= 167)

♦ Received allocated intervention (n= 167)

♦ Did not receive allocated intervention (give reasons) (n=/ )

Lost to follow-up (give reasons) (n= )

Discontinued intervention (give reasons) (n= )

Allocated to intervention (n= 167)

♦ Received allocated intervention (n= 167)

♦ Did not receive allocated intervention (give reasons) (n=/ )

Analysed (n= 165)
♦ Excluded from analysis (give reasons) (n=2, premature termination of experiment, artificial data)

Randomized (n= 167)
